# Protein Profiling of a Cellular Model of NAFLD by Advanced Bioanalytical Approaches

**DOI:** 10.3390/ijms23169025

**Published:** 2022-08-12

**Authors:** Alessandra Anna Altomare, Gilda Aiello, Jessica Leite Garcia, Giulia Garrone, Beatrice Zoanni, Marina Carini, Giancarlo Aldini, Alfonsina D’Amato

**Affiliations:** 1 Department of Pharmaceutical Sciences, University of Milan, Via L. Mangiagalli 25, 20133 Milan, Italy; 2 Department of Human Science and Quality of Life Promotion, Telematic University San Raffaele, 00166 Rome, Italy; 3 Medical School, Sao Paulo State University, Botucatu 22250-020, Brazil; 4 Unitech OMICs Platform, University of Milan, Viale Ortles 22/4, 20139 Milan, Italy

**Keywords:** nano liquid chromatography, tandem mass spectrometry, proteins, signaling, NAFLD

## Abstract

Advanced quantitative bioanalytical approaches in combination with network analyses allow us to answer complex biological questions, such as the description of changes in protein profiles under disease conditions or upon treatment with drugs. In the present work, three quantitative proteomic approaches—either based on labelling or not—in combination with network analyses were applied to a new in vitro cellular model of nonalcoholic fatty liver disease (NAFLD) for the first time. This disease is characterized by the accumulation of lipids, inflammation, fibrosis, and insulin resistance. Hepatic G2 cells were used as model, and NAFLD was induced by a complex of oleic acid and bovine albumin. The development of the disease was verified by lipid vesicle staining and by the increase in the expression of perilipin-2—a protein constitutively present in the vesicles during NAFLD. The nLC–MS/MS analyses of peptide samples obtained from three different proteomic approaches resulted in accurate and reproducible quantitative data of protein fold-change expressed in NAFLD versus control cells. The differentially regulated proteins were used to evaluate the involved and statistically enriched pathways. Network analyses highlighted several functional and disease modules affected by NAFLD, such as inflammation, oxidative stress defense, cell proliferation, and ferroptosis. Each quantitative approach allowed the identification of similar modulated pathways. The combination of the three approaches improved the power of statistical network analyses by increasing the number of involved proteins and their fold-change. In conclusion, the application of advanced bioanalytical approaches in combination with pathway analyses allows the in-depth and accurate description of the protein profile of an in vitro cellular model of NAFLD by using high-resolution quantitative mass spectrometry data. This model could be extremely useful in the discovery of new drugs to modulate the equilibrium NAFLD health state.

## 1. Introduction

Nonalcoholic fatty liver disease (NAFLD), characterized by ectopic lipid storage in hepatocytes not driven by alcohol abuse, is the most common form of liver disease, affecting around 24% of individuals globally [1]. NAFLD has been associated with changes in cytokine profiles, insulin resistance, and dysfunctional lipid transport, but it is not fully known to what extent these factors initiate damage or are markers of damage [2]. Experimental models, whether using animals or in vitro, are widely used to study NAFLD, and are crucial to understanding its pathogenesis and hepatocellular consequences, as well as to evaluate potential pharmacological therapies. Human hepatocellular carcinoma cells (HepG2) and the induction of lipid accumulation by oleic acid (OA) have been reported as a model of hepatic steatosis [3,4,5,6]. OA is a monosaturated omega-9 fatty acid (C18:1), present in the diet and end products of de novo fatty acid synthesis, and abundantly found in steatotic livers [7,8]. Thus, excess OA is associated with hepatic steatosis in humans. In addition, OA-induced steatosis causes changes in cell morphology; increases tumor necrosis factor alpha (TNF-α) levels, lipid peroxidation, and apoptosis; and decreases expression of peroxisome proliferator-activated receptor alpha (PPAR-α) [9,10,11,12]. Nevertheless, the underlying molecular mechanisms behind the onset of steatosis and the steatosis-induced alteration of the proteome in an in vitro hepatic cellular model have not been deeply explored. The advent of mass-spectrometry-based proteomics and bioinformatics has led to a comprehensive understanding of the molecular mechanisms under physiological and pathological conditions. To date, several methodological strategies, including specific sample labelling approaches and fractionation methods, have been used to increase throughput, with higher capacity of peptide sequencing and identification to provide more accurate data. Label-free quantitative methods are considered to be the most straightforward laboratory approach, since samples can be measured without resorting to complex preparation protocols. However, labelled approaches, which require additional pretreatment (i.e., metabolic (SILAC) or chemical (TMT)), have some important advantages that make them the methods of choice for specific experimental designs. Firstly, in label-free experiments, each sample is measured in a separate mass spectrometry (MS) run, whereas in labelled experiments, samples from each condition are combined before the MS run, reducing the machine time required. Another disadvantage of label-free approaches is that the analyses are affected by sample-to-sample variations due to experimental measurement conditions (e.g., temperature, experimenter, column condition)—differences that do not occur in labelled experiments because the compared samples are measured in the same MS run. Conversely, an advantage of label-free experiments is that any sample can be directly compared with any other, making the technique easily adaptable to the study’s needs, whereas in labelled experiments it is only possible to compare samples that have been mixed previously and measured in a single run. Overall, it is necessary to consider the advantages and disadvantages in order to find the right balance in the choice of approach. Three complementary quantification strategies used for proteomics—label-free (LF) [13], stable isotope labelling by amino acids in cell culture (SILAC) [14], and isobaric tandem mass tags (TMT) [15]—were employed here. Although several publications address the comparison of quantification methods in a pairwise manner [16], the comprehensive quantitative data obtained using three different analytical strategies showed the ability to deeply describe an in vitro NAFLD cellular model for the first time. Exploiting the high potential of high-resolution mass spectrometers such as the Orbitrap Fusion, coupled with network analyses, the comprehensive characterization of the proteome dynamics of steatotic HepG2 cells was assessed. Statistical evaluations were performed to reveal proteins with significantly different expression profiles, which were finally ranked according to their significance and fold-changes. Through a bioanalytical approach, the integration of quantitative data derived from the LF, SILAC, and TMT advanced proteomic technologies was conducted for a deeper investigation of the HepG2 cell proteome associated with induced steatosis. The model and the quantitative approach can be applied to test new drugs in NAFLD, identifying the main target, the mechanism of action, and the level of toxicity.

## 2. Results and Discussion

### 2.1. Oleic-Acid-Induced NAFLD in HepG2 Cells: Validation of the Model

**Figure 1:**
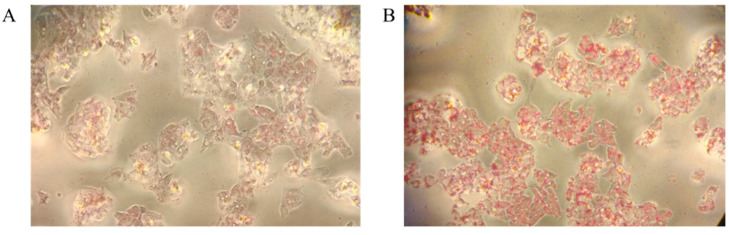
(**A**) Oil red O staining for the detection of lipid accumulation in induced steatotic HepG2 (20× optical zoom). (**B**) Effect of oleic acid on TG content HepG2; the TG content in the cells increased significantly after the treatment with 0.6 mM OA compared with the control group.

**Figure 2:**
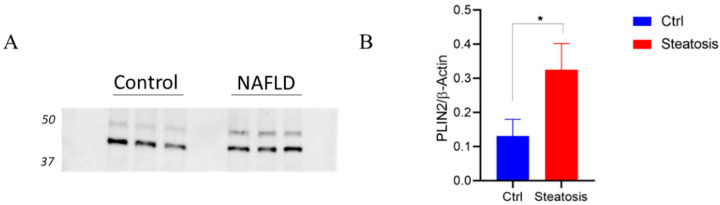
(**A**) Western blot analyses of perilipin-2 (upper band) in NAFLD versus control. (**B**) The signals of beta-actin (lower band) were used to normalize the amounts of loaded protein samples in the densitometric analyses. * indicates a *p* value < 0.05.

To induce steatosis in the hepatic G2 cellular model and obtain an in vitro model of nonalcoholic fatty liver disease (NAFLD), different methods based on oleic acid were adopted [17,18]. The accumulation of lipids was followed by Oil Red O staining. As shown in, Figure 1 the most effective approach was based on the use of a complex of OA (0.6 mM) and bovine serum albumin (BSA) at a 5:1 ratio (OA:BSA) added to the cellular medium for 24 h. The increasing in TG staining in the steatotic state (Figure 1B) versus control (Figure 1A) demonstrates the accumulation of lipid droplets (red color) and the induction of NAFLD. TG content was also measured using a TG assay kit. To further confirm the induction of steatosis, perilipin-2 (PLIN-2)—a structural component of lipid droplets, involved in the formation and maintenance of lipid storage droplets—was detected by Western blotting and quantified by densitometric analyses (Figure 2A,B). The increase in PLIN-2 in the steatotic model versus the control was 2.4-fold, attesting to the accumulation of lipid droplets and the induction of NAFLD.

### 2.2. Description of the NAFLD Proteome by Quantitative Approaches: LFQ, SILAC, and TMT

**Figure 3:**
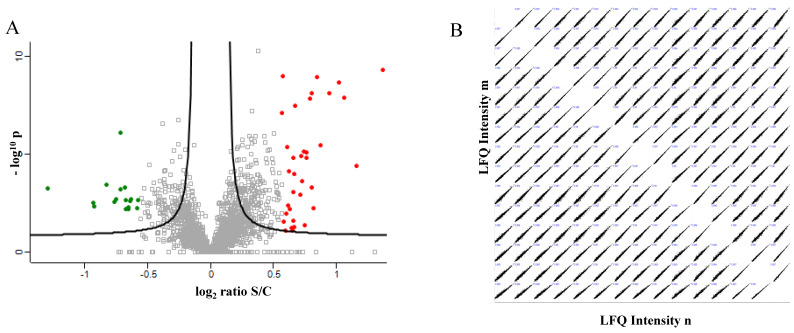
(**A**) Differentially regulated proteins in NAFLD (S), compared with control (C) resulted from label free analysis. Green represents downregulated proteins (log2 ratio ≤ −0.5). Red represents upregulated proteins (log2 ratio ≥ 0.5). (**B**) Correlation analyses of the LFQ intensity of 2 biological and 3 technical replicates. Pearson’s correlation coefficient ≥ 0.9.

The present study shows the advantages of the combined use of label-free (LF) and label-based strategies such as SILAC and TMT to deeply unravel the proteome of nonalcoholic steatohepatitis-induced HepG2 cells. In the label-free proteomic approach, the extracted proteins from control and steatotic cells (two biological and three technical replicates) were quantified by colorimetric assay and digested in solution by trypsin (ratio 1:20). The peptide mixture was purified and concentrated using zip tips and then directly analyzed by nLC–MS/MS. The raw data analyzed using MaxQuant and Perseus software resulted in the quantification of 2482 proteins, of which 17 down- and 36 upregulated proteins with a fold-change greater than 1.5 were shown in the volcano plot (Figure 3A); green spots represent downregulated proteins, whereas red spots represent upregulated proteins. The correlation analyses of LFQ intensity resulted in a Pearson’s correlation coefficient higher than 0.9, highlighting the reproducibility of the three technical replicates of each sample in biological duplicates (Figure 3B).

**Figure 4:**
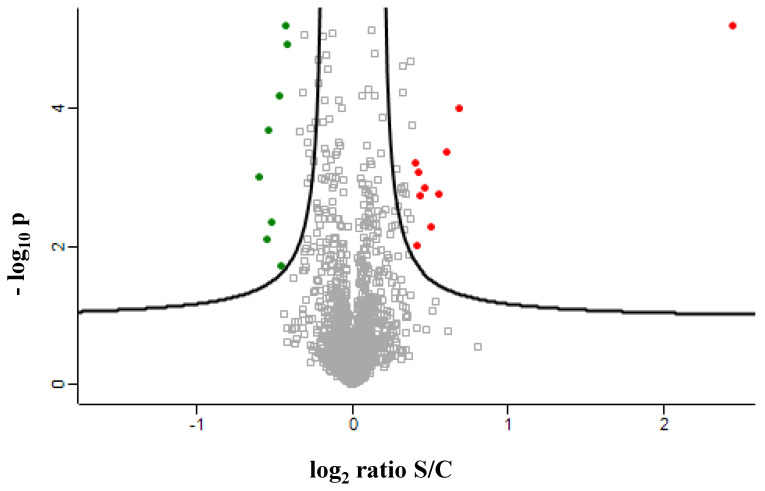
Differentially regulated proteins in NAFLD (S), compared with control (C) resulted from SILAC analysis. Two biological and three technical triplicates.

The SILAC experiment relied on HepG2 cells being metabolically labelled using media containing heavy Arg10 [^13^C_6_,^15^N_4_] L-arginine and heavy Lys8 [^13^C_6_/^15^N_2_] L-lysine (Lys8), or normal Arg0 and Lys0. The steatotic cells (heavy) were mixed 1:1 with control cells (light) by cell counting. The extracted proteins in 8 M urea were digested in solution by trypsin and analyzed by nLC–MS/MS in triplicate. The incorporation of heavy amino acids was tested after eight doublings of the cells using a 1:1 mixture of heavy control cells and light control cells. The incorporation was almost complete (around 99%), as verified by the two-sided *t*-test of peptide intensities. The two groups—light and heavy—did not show any differences (data not shown). Three technical replicates of each mixture prepared in biological duplicates successfully led to the quantification of 1906 proteins on the basis of the normalized heavy/light ratio, of which there were 10 up- and 8 downregulated proteins in steatotic (heavy) cells versus control (light) cells, with a fold-change of 1.3 (Figure 4).

**Figure 5:**
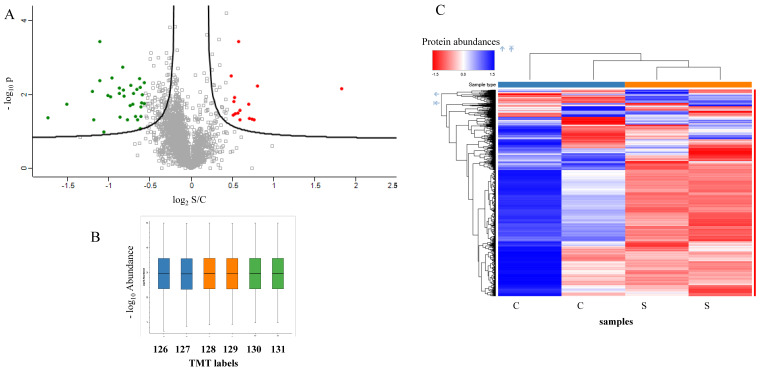
(**A**) Differentially regulated proteins in NAFLD (S), compared with control (C) resulted from TMT analyses; biological duplicates and technical triplicates. (**B**) Correlation analyses of the abundances of different samples labelled by TMT. (**C**) Heatmap of 2 controls and 2 NAFLD samples.

The tandem mass tag (TMT) quantitative proteomic labelling approach was applied using a six-plex of molecules, containing the MS/MS-reported ions of 126, 127, 128, 129, 130, and 131 m/z. The extracted proteins from steatosis (n = 2) and control cells (n = 2) were quantified and digested by trypsin. The peptides were then labelled using the six molecules according to the manufacturer’s protocol. The complete six-plex labelled peptide mix was fractionated by high-pH chromatography on the tip, and the 10 fractions were analyzed by nLC–MS/MS in duplicate. In total, 3567 proteins were quantified, of which there were 14 up- and 33 downregulated proteins in steatotic versus control samples (Figure 5A). The normalized abundances of the peptides modified by the different molecules were comparable (Figure 5B). The heatmap of protein abundances showed a clear clustering of the samples belonging to the steatotic and control groups (Figure 5C). The fractionation step not only allowed an accurate quantification of the six reported ions in the fragmentation spectra, simplifying the analyzed sample, but also allowed a deeper description of the proteome, significantly increasing the number of quantified proteins. The complete list of quantified proteins obtained using MaxQuant software in each approach is reported in Supplementary Table S1.

**Figure 6:**
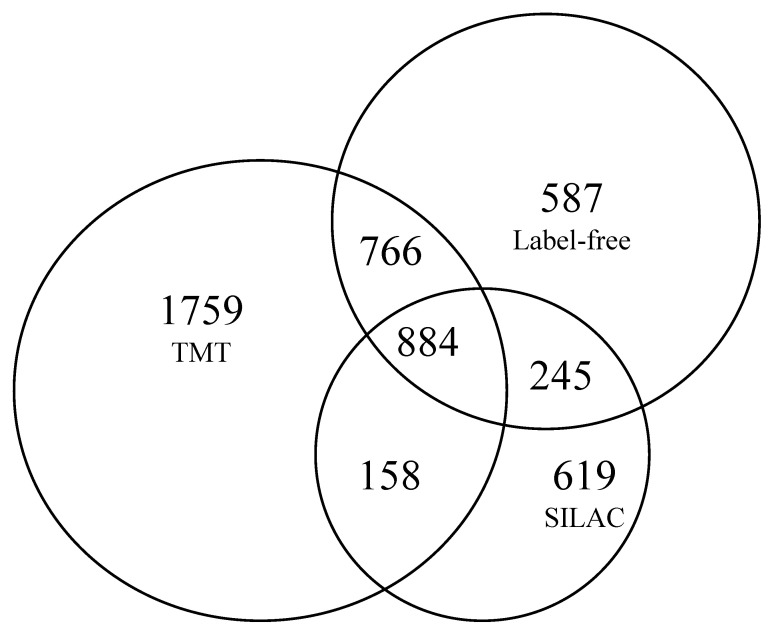
Comparison of quantitative results obtained from different quantification approaches by Venn diagram.

Overall, the three strategies allowed us to deeply explore the proteome; 4086 proteins were quantified, 884 of which were common to all three quantitative proteomic approaches, whereas 1042 proteins were common among the two labelling strategies, i.e., TMT and SILAC. The label-free technique shared 1650 quantified proteins with the TMT experiment and 1129 with the SILAC experiment (Figure 6).

Several proteins were mostly dysregulated in NAFLD HepG2 cells (Table 1 and Supplementary Table S1), including the lipid-droplet-associated hydrolase LDAH belonging to the AB hydrolase superfamily, which is a serine lipid hydrolase associated with lipid droplets, and was found to be upregulated (log_2_ ratio = 0.60). Being highly expressed in macrophage-rich areas in atherosclerotic lesions, it promotes cholesterol ester turnover in macrophages [19,20]. Additionally, perilipin-2 (PLIN-2, ADFP), involved in the development and maintenance of adipose tissue, was found to be upregulated here (log_2_ ratio = 2.44) (Table 1), as also confirmed by Western blot analyses (Figure 2) [10]. PLIN-2 binds and sequesters lipids at the lipid droplet surface, and enhances lipid transport, suggesting that it may act as a regulatory protein, governing the release and deposition of lipids, including eicosanoids that are stored in lipid droplets [21]. Conversely, liver carboxylesterase 1 (CES1), which plays a regulatory role in hepatic fat metabolism, was found to be downregulated (log_2_ ratio = −0.36). CES1/Es-x-knockout mice presented increased hepatic lipogenesis and oversecretion of apolipoprotein B (apoB)-containing lipoproteins (very low-density hepatic lipoproteins), leading to hyperlipidemia and increased fat deposition in peripheral tissues [20]. Connected to the mitochondrial activity, prohibitin-2 (PHB2, log_2_ ratio = 0.89) was altered in HepG2. Prohibitins are novel receptors of inner-membrane mitophagy, acting as proteins or even lipid scaffolds that may offer another means to ensure the functional integrity of the inner membrane.

### 2.3. Description of the NAFLD Proteome by Network Analyses Using Quantitative Data

Network analyses described the main functional and disease modules involved during the induction of NAFLD in HepG2 cells, based on complete quantitative data. Several canonical pathways were differentially expressed, such as “Unfolded protein response” (six genes, *p*-value = 1.32 × 10^−6^), which was negatively induced (z-score = −2.44), and “Protein Kinase A signaling” (nine genes, *p*-value = 9.12 × 10^−6^), which was also negatively induced (z-score = −2.33) (Table 2). “Ferroptosis signaling pathway” (six genes, *p*-value = 1.38 × 10^−4^) was induced positively (z-score = 1.63). Ferroptosis is an iron-dependent cell death function, characterized by an accumulation of lipid peroxides and induced by the failure of glutathione enzymes involved in antioxidant defense [22]. The detoxicant enzymes involved in the NRF2 pathway—glutathione S-transferase zeta 1 and microsomal glutathione S-transferase 1—were downregulated (log_2_ ratio = −0.47 and −0.62, respectively), confirming the implications of the ferroptosis pathway. In addition, hepatic ferroptosis triggers inflammation in nonalcoholic steatohepatitis, leading to liver damage, infiltration of immune cells, and inflammatory reaction. Proteins involved in the activation of the ferroptosis pathway included transferrin receptor protein 1 (TFRC, log_2_ ratio = 0.6) and zinc transporter ZIP14 (SLC39A14, log_2_ ratio = 0.40), which mediate the iron uptake by directly transporting non-transferrin-bound iron protein (NTBI) across the cell membrane. as Additionally, squalene synthase (FDFT1, log_2_ ratio = 0.40), involved in the first step of cholesterol biosynthesis, was found to be altered [23]. The dysregulation of ferroptosis decreases hepatic expression of glutathione peroxidase 4 (GPX4) and, conversely, increases 12/15-lipoxygenase in NASH-related lipid peroxidation and its associated cell death [24]. The “hepatic fibrosis signaling pathway” (seven genes, *p*-value = 9.55 × 10^−3^) was upregulated (z-score = 1.13). Hepatic fibrosis is one of the hallmarks of NAFLD, which is characterized by extensive accumulation of connective tissue following extensive tissue damage [25]. The ECM in the liver plays a major role in the overall microenvironment for hepatocytes as well as other hepatic cells. The hepatic ECM responds with continuous remodeling of the matrix by inducing excessive accumulation of matrix proteins, such as collagens, fibronectins, and laminins, as well as proteoglycans [26]. The modulation of “Actin Cytoskeleton Signaling” (five genes, z-score = 0.45, *p*-value = 1.38 × 10^−2^) was observed in the steatosis model. Myosin-10 (MYH10, log_2_ ratio = 0.62), myosin regulatory light chain 12A (MYL12A, log_2_ ratio = 0.55), and non-muscle myosin heavy chain 9 (MYH9, log_2_ ratio = 0.42) were involved in the modulation of actin signaling (Table 1).

**Figure 7:**
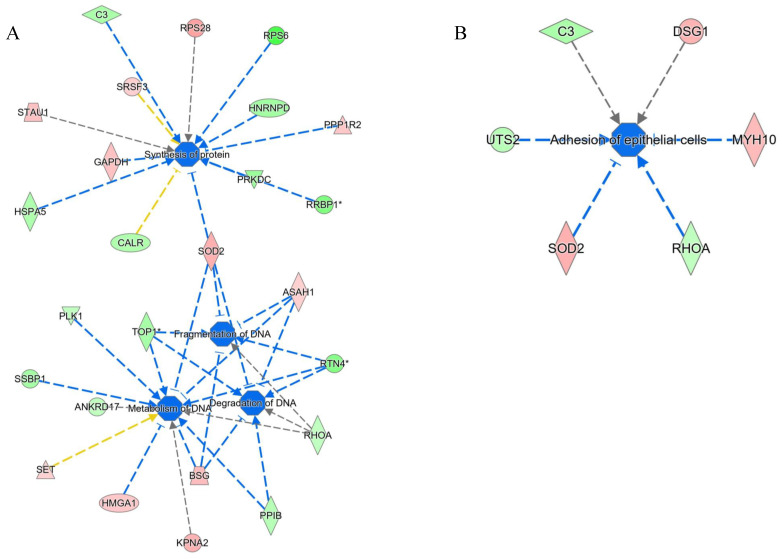
Negatively induced pathways (blue hubs) in NAFLD versus control cells: (**A**) Synthesis of proteins and DNA metabolism. (**B**) Adhesion of epithelial cell pathways. The intensity of the color is positively related to the up- (red) or downregulation (green) of genes; orange lines lead to activation, blue lines lead to deactivation, yellow lines represent findings inconsistent with the state of the downstream molecule, and gray lines represent effects that were not predicted. The * indicates the presence of protein isoforms.

**Figure 8:**
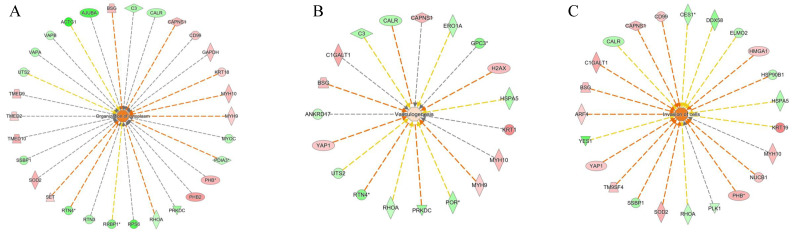
Positively induced pathways (orange hubs) in NAFLD versus control cells: (**A**) organization of cytoplasm, (**B**) vasculogenesis, and (**C**) invasion of cell pathway. The * indicates the presence of protein isoforms.

Network analyses also resulted in the modulation of functional and disease modules, such as the negatively induced “synthesis of proteins”, “DNA metabolisms”, and “adhesion of epithelial cell” (Figure 7). “Organization of cytoplasm”, “vasculogenesis”, and “invasion of cell pathways” were positively induced (Figure 8). The presence of fibrosis is supported by the downregulation of RHOA (log_2_ ratio = −0.42), which is involved in the renin–angiotensin system, playing an important role in the onset of hepatic fibrosis and steatosis [27]. However, the role of RHOA is also connected to the regulation of cell shape, polarity, and locomotion, exercising its main effects on actin polymerization, actomyosin contractility, and cell adhesion. Specifically, RHOA plays a key role in regulating the integrity of cell–extracellular matrix and cell–cell adhesions, the latter including both adherens junctions and tight junctions [28].

## 3. Materials and Methods

### 3.1. Reagents

Acetonitrile and 0.1% formic acid in water (FA) were of analytical grade for LC–MS analysis, and were obtained from Carlo Erba Reagents (Milano, Italy). Trypsin (proteomics grade, product number T7575-1KT), iodoacetamide (IAA, BioUltra, product number I1149), DL-dithiothreitol (DTT for HPLC, ≥98.0%, product number D0632), trifluoroacetic acid (TFA for HPLC, ≥99.0%, product number 411543), hydroxylamine 50 wt% in H_2_O, (99.999%, product number 467804), and triethylammonium bicarbonate 1.0 M, pH 8.5 ± 0.1 (TEAB, product number T7408), were purchased from Merck Life Science S.R.L. (Milano, Italy), while Tris (2-carboxyethyl) phosphine (TCEP neutral pH, product number 77720) was purchased from Life Technologies Italia (Monza, Italy). Trimethylamine (TEA), Acclaim PepMap C18 columns, and EASY-Spray columns were purchased from Thermo Scientific, (Rodano, Italy). All solutions were in water. Arg10 [^13^C_6_,^15^N_4_] L-arginine, Lys8 [^13^C_6_/^15^N_2_] L-lysine (Lys8), normal Arg0 and Lys0, DMEM media without Arg and Lys, and dialyzed serum were purchased from Thermo Scientific (Rodano, Italy).

### 3.2. Cell Culture

HepG2 cells were grown in Dulbecco’s modified Eagle’s medium (DMEM) supplemented with 10% fetal bovine serum (FBS) (Gibco^®^,Sigma-Aldrich, Burlington, MA, USA), 2 mM L-glutamine (Gibco^®^), and 1 mM sodium pyruvate (Gibco^®^), and maintained in incubator at 37 °C and a 5% CO_2_ atmosphere. The cells were detached from the culture flasks with trypsin–EDTA solution (Lonza Bioscience, Rome, Italy) for 5 min when reaching 70–80% confluence. The cell viability was determined using the trypan blue 0.4% (Sigma-Aldrich, Burlington, MA, USA) exclusion assay. For the SILAC experiment, HepG2 cells were grown in two DMEM media—heavy (containing Lys8 and Arg10) and light (containing Lys0 and Arg0)—for 8 doublings before starting the NAFLD induction.

### 3.3. Induction of Steatosis

To promote the accumulation of lipids, HepG2 cells were incubated under exposure to oleic acid (OA) for 24 h, as described by Fujimoto et al. [17], to induce the formation of lipid vesicles. A complex of OA and bovine serum albumin (BSA) was prepared at a 5:1 ratio (OA:BSA), with a final concentration of 0.6 mM OA (O1008, Sigma-Aldrich). The complex was produced in the medium 30 min before being added to the cells. The control conditions were maintained in complete medium without the OA:BSA complex.

### 3.4. Oil Red O Staining and Intracellular Lipid Content Assay

HepG2 cells (3 × 10^5^/well) were grown in 6-well plates in triplicate for 24 h. Then, the cells were incubated for 24 h under control and steatosis conditions. After this, the media were aspirated and the cells were washed with warmed PBS and fixed with 4% paraformaldehyde for 10 min. The cells were then gently washed again with 60% isopropanol for 1 min. The Oil Red O staining (Sigma-Aldrich) solution was added for 30 min [18,19]. Finally, the cells were washed 4 times with deionized water and then allowed to dry. The images were captured from random points. Intracellular lipid content was determined using the Triglyceride Quantification Kit (MAK266, Sigma-Aldrich). HepG2 cells were grown and steatosis was induced as previously described. The experiment was performed in triplicate for each condition (control and steatosis). Then, the cells were detached from the culture flasks, counted, and their viability was checked. Sample preparation and measurements were performed as indicated by the kit protocol.

### 3.5. Western Blots and Densitometric Analyses

For the immunoblot analysis, 5 µg each of control and steatosis proteins were separated by SDS–PAGE in reducing conditions. Proteins were dissolved in 30 µL of 2× Laemmli Sample Buffer (100 mM DTT). The samples were denatured, incubating for 5 min at 95 °C. Control and steatosis samples were separated on Any KD™ Mini Protean^®^ TGX™ precast gels. The proteins were then transferred onto nitrocellulose membranes using the Bio-Rad Trans-Blot Turbo Transfer System (Bio-Rad, Hercules, CA, USA). Blotted membranes were incubated in Ponceau S Staining Solution (0.5% *w*/*v* in 1% *v*/*v* acetic acid) for 5 min at room temperature, and then washed in water until distinct reddish-pink protein bands were visible (1–5 min). Images were acquired using the ChemiDoc MP Imaging System (Bio-Rad, Hercules, CA, USA), and subsequently washed in 1× Tris-buffered saline (TBS) with 0.2% Tween (TBST) three times for 5 min at room temperature until the protein bands were no longer visible. After incubation of the blotted membranes with dried 5% fat milk in TBST overnight at 4 °C, the membrane was washed 3 times (5 min) in TBST and then incubated for 1 h at room temperature with the primary antibody—murine monoclonal anti-perilipin-2 antibody ((N-terminus) murine monoclonal antibody, 1:100, AP125 (Cat. No. 690102), PROGEN Biotechnik GmbH, Heidelberg, Germany). The membrane was washed as previously described and then incubated with the secondary antibody—anti-mouse IgG H&L–HRP (1:10,000, ab205719, Abcam, Cambridge, UK)—for 1 h. The membrane, after 5 washing steps in TBST, was incubated with β-actin ((8H10D10) murine mAb (HRP conjugate), 1:1000). After 5 washing steps in TBST, chemiluminescence detection was performed using SuperSignal West Atto Substrate (Thermo Scientific, Rodano, Italy) at room temperature; ECL Western blot detection reagent was added for 30 s, and the signal was acquired (3 s exposure in Chem High-sensitivity mode), placing the blotting membrane back on the sample stage of the ChemiDoc MP Imaging System (Bio-Rad, Hercules, CA, USA) and elaborating the image using Image Lab 4.1 software (Bio-Rad, Hercules, CA, USA).

### 3.6. Protein Extraction, In-Solution Trypsin Digestion, and TMT Labeling

The cell pellets of 2 biological replicates of the control and NAFLD samples were resuspended in the solubilization buffer (8 M urea in 50 mM TEAB, 30 mM NaCl, pH 8.5, and 1% protease inhibitor) and incubated on ice for 5–10 min. The cell lysates were further homogenized by sonication in an ice bath three times each for 15 s, with 1 min intervals, using an ultrasonicator. Samples were centrifuged at 14,000 rpm for 20 min at 4 °C. The protein supernatant was collected in a new tube, and pelleted cell debris was discarded. Samples were stored at −80 °C until they were used for further experiments. The protein estimation was carried out by using the BCA assay; 10 µg of protein was diluted in 50 mM TEAB and then reduced with 5 mM TCEP for 30 min at 52 °C, before being centrifuged at 500 rpm and alkylated with 15 mM iodoacetamide (Merck Life Science S.r.l., Milan, Italy, Sigma-Aldrich) for 20 min in the dark at room temperature. The trypsin digestion was performed at a 1:20 enzyme/protein ratio (*w*/*w*) (sequencing-grade trypsin; Roche, Monza, Italy) overnight at 37 °C. The samples were labeled using the 6-plex TMT kit (Thermo Fisher Scientific, product no. 90061, lot no. UH287619) and derivatized. Briefly, 0.8 mg of each TMT reagent was dissolved in 41 μL of anhydrous acetonitrile. Carefully, 41 µL of the TMT Label Reagent was added to each 100 µL sample. After 1 h, the reaction was quenched with 8 μL of 5% hydroxylamine for 15 min. After labeling, the peptide solutions were combined according to the batch arrangement. To increase the number of peptide identifications, the labeled peptides were fractionated using the Thermo Scientific™ Pierce™ High-pH Reversed-Phase Peptide Fractionation Kit (Product No. 84868). To enhance the depth of the discovery proteome, these samples were subjected to high-pH fractionation as described elsewhere (Pub. No. MAN0015701, Pub. Part No. 2162566.1 Rev A.0). A mixture of TMT-labeled peptides (90 μg) was dissolved in 300 μL of 0.1% TFA. In total, 8 fractions were collected with an increased % of acetonitrile in 0.1% trimethylamine (TEA). Each sample tube was evaporated to dryness using a SpeedVac concentrator. Dry samples were resuspended in an appropriate volume (40 µL) of 0.1% FA before LC–MS analysis.

### 3.7. Protein Extraction and In-Solution Trypsin Digestion of SILAC and Label-Free Samples

HepG2 cells (SILAC-labeled and unlabeled, with 2 biological replicates each of control and NAFLD samples) were trypsinized and pelleted by two cycles of centrifugation at 400× *g* and room temperature for 5 min. The whole protein was extracted by using a buffer composed of 8 M urea in 50 mM Tris-HCl, 30 mM NaCl at 8.5 pH, and 1% protease inhibitor cocktail (Sigma-Aldrich), followed by centrifugation at 14,000× *g* and 4° C for 30 min. Then, 20 µg of the proteins in 50 mM NH₄HCO₃ was reduced with 5 mM DL-dithiothreitol for 30 min at 52 °C, and then centrifuged at 500 rpm and alkylated with 15 mM iodoacetamide for 20 min in the dark at room temperature. The trypsin digestion was performed at a 1:20 enzyme/protein ratio (*w*/*w*) overnight at 37 °C.

### 3.8. High-Resolution Quantitative Liquid Chromatography with Tandem Mass Spectrometry (LC–MS/MS) Analysis

The analysis was performed using a Dionex Ultimate 3000 nano-LC system connected to an Orbitrap Fusion™ Tribrid™ Mass Spectrometer equipped with a nano-electrospray ion source (Thermo Fisher Scientific). Tryptic peptides were separated on an EASY-Spray 50 cm × 75 μm ID column packed with Thermo Scientific Acclaim PepMap RSLC C18 column (3 μm, 100 Å particles). The mobile phases were 0.1% formic acid in water (Solvent A) and 0.1% formic acid in water/acetonitrile, 2:8/*V*:*V* (Solvent B). The elution gradient consisted of 4–28% B for 90 min and then 28–40% for 10 min, followed by 95% for the next 6 min to rinse the column. The column was then re-equilibrated for 20 min. The total runtime was 130 min. The flow rate was 300 nL/min, and the temperature was set to 35 °C. Each sample was analyzed in three technical replicates. A blank sample was injected after each triplicate to avoid sample carryover. The operational m/z range to obtain MS spectra was set to 375–1500 Da at 120,000 resolution. The collision energy was set to 35 eV, and the system operated in a data-dependent mode, with 3 s between each master scan. The TMT-labeled samples were analyzed by MS/MS, and quantitative analyses were performed from MS3 scans on reporter ions. An equal volume of each high-pH peptide fraction was first resuspended in 0.1% FA. The samples were analyzed at UNITECH OMICs (University of Milano, Italy) using a Dionex Ultimate 3000 nano-LC system (Sunnyvale, CA, USA) connected to an Orbitrap Fusion™ Tribrid™ Mass Spectrometer (Thermo Scientific, Bremen, Germany) and equipped with a nESI ion source. Peptide mixtures were pre-concentrated onto an Acclaim PepMap C18 column (5 µm, 100 Å, 100 µm ID × 2 cm), and separated at 35 °C on an EASY-Spray PepMap RSLC C18 column (3 µm, 100 Å, 75 µm ID × 50 cm). Elutions were run in gradient mode from 96% mobile phase A (0.1% FA) to 60% mobile phase B (0.1% FA/acetonitrile, 20/80, *v*/*v*). The total gradient was 110 min, the flow rate was 300 nL/min, and the total runtime was 150 min. MS spectra were collected in positive ion mode, in the data-dependent (DDA) synchronous precursor selection (SPS) MS/MS/MS (MS3) mode. The peptides were ionized with a spray voltage of 1700 kV. The instrumental method included Orbitrap MS1 scans (resolution of 120,000; mass range of 375–1500 m/z; automatic gain control (AGC) target 4 × 10^5^, maximum injection time of 50 ms). During the MS2 analyses, the precursor ions were filtered according to the charge state (required > 1 z), dynamic exclusion (60 s with a ± 10 ppm window), and monoisotopic precursor selection. Precursors were isolated in quadrupole mode using a width of 0.7 m/z, and were fragmented by collision-induced dissociation (CID), followed by ion trap MS2 scans (CID collision energy of 35%; AGC target 1 × 10^4^; turbo ion trap scan rate; maximum injection time of 50 ms). Quantitative SPS-MS3 scans operating in data-dependent mode were selected, with a precursor selection range of 400–1200 m/z and 10 SPS precursors. For the MS3 scan, the MS1 precursor was isolated using a 2 m/z wide window (resolution of 30,000; HCD collision energy of 65%; scan range of 100−500 m/z; AGC target 5 × 10^4^; maximum injection time of 54 ms).

### 3.9. Data Analysis: Protein Quantification and Statistics

The raw files from the LF, SILAC, and TMT experiments were separately processed using the MaxQuant proteomics software package (version V1.6.6.0), including the Perseus software platform (version 1.6.2.1, Max-Planck-Gesellschaft, München, Germany) for statistics. The MS/MS spectra (raw files) were searched against a merged protein sequence database consisting of a human proteome database (20,154 reviewed sequences). The settings used included fixed modifications for carbamidomethyl (C), and variable modifications for oxidized methionine (M) and acetyl (N-terminus). High-confidence and unique peptides (minimum 1 peptide per protein at a PSM FDR of 0.01 and protein FDR of 0.01) were used for protein identification. Further parameters were set as follows: first and main search peptide tolerance = 20 and 4.5 ppm, respectively; isotope and centroid match tolerance = 2 and 8 ppm, respectively; maximum number of missed cleavages = 2. For LFQ data analysis, the match-between-runs (MBR) option was activated, with the match time window set to 0.7 min and the initial alignment window to 20 min. For TMT data analyses, the multiplex type was selected, and the correction factors suggested by the manufacturer were inserted. For the SILAC data analyses Lys8 and Arg10 were selected as heavy amino-acidic residues. Only unique peptides were selected for quantification, with a minimum ratio count of 1.

### 3.10. Network Analysis

To identify the biological processes, diseases, and functions associated with the differentially expressed proteins in the experimental groups, data were analyzed using the Ingenuity Pathway Analysis software (latest release; QIAGEN, Milan, Italy) based on the Gene Ontology database. The statistical enrichment of the involved pathways was performed using the right-tailed Fisher’s exact test, in correlation with QIAGEN Knowledge Base, assigning a *p*-value (https://digitalinsights.qiagen.com/products/features/analysis-match/, accessed on 2021). The significance indicates the probability of the association of molecules from the experimental dataset with the pathway by random chance alone. The overall activation/inhibition states of canonical pathways were predicted based on a z-score algorithm. The pathways were colored to indicate their activation z-scores: orange predicts a gain of function, while blue predicts a loss of function.

## 4. Conclusions

The cellular NAFLD model was induced in vitro and described by advanced bioanalytical approaches for the first time. The complex of oleic acid and BSA was able to induce the accumulation of lipids in vesicles characterized by perilipin-2, the expression of which was greatly increased in HepG2 cells. The NAFLD cellular model was deeply described by advanced network analyses using quantitative data on protein expression. Three orthogonal quantitative proteomics approaches were used to obtain a more complete protein profiling and identification of a higher number of differentially expressed proteins after the induction of NAFLD. In general, the LFQ approach achieved a high proteome coverage, but was outperformed by label-based approaches in terms of the total numbers of significantly quantified gene products. By comparing the two labelled approaches used, we found that the TMT experiment achieved the maximum proteome coverage, and was more efficient in the identification of up- and downregulated pathways. This outstanding performance may also be explained by considering the advantages promoted by the fractionation protocol applied, which significantly amplified the proteome coverage. In contrast, the SILAC experiment showed the lowest coverage and the highest number of missing values. In addition to the different performances, considering the number of quantified proteins, each quantitative approach identified similar modulated pathways. This interesting technical aspect attests that there is no method of choice for analyzing cellular signaling in cell culture models that depend on the experimental design of the study, on the number of samples to be tested, and on the instrumental platform at our disposal.

The complementary analyses in combination with the statistically enriched protein network enabled the identification of several functional and disease modules involved in NAFLD, with highly significant *p*-values and z-scores. The main negatively induced modules were related to metabolic pathways such as “protein synthesis” and “DNA metabolism”. The downregulation of “cell-cell adhesion pathways” corresponded to the positive induction of the “organization of cytoplasm” and “invasion of cell” pathways, attesting to the increase in cellular proliferation due to inflammation or impairment of oxidative stress defense, highlighted by the downregulation of glutathione enzymes. In conclusion, the advanced and comprehensive bioanalytical approach allows the accurate description of the biological complex matrix by using quantitative mass spectrometry data. The described comprehensive approach has the potential to be applied for in vitro disease or cellular health modeling before and upon treatment with new drugs.

## Figures and Tables

**Table 1:** Quantified proteins in NAFLD versus control samples obtained via three approaches.

Log2 Ratio	Protein Accession Numbers	Protein Names	Gene Names	Unique Peptides	Molecular Weight (Da)
2.44	Q6FHZ7	Perilipin-2	ADFP	14	48,075
1.83	Q53GP1	N-sulfoglucosamine sulfohydrolase	SGSH	2	56,642
1.09	P81605	Dermcidin	DCD	4	11,284
0.89	J3KPX7	Prohibitin-2	PHB2	8	33,239
0.83	B2R4R9	40S ribosomal protein S28	RPS28	2	78,409
0.81	Q9H477	Ribokinase	RBKS	2	34,143
0.76	A0A024RA32	Glycoprotein-N-acetylgalactosamine 3-beta-galactosyltransferase 1	C1GALT1	2	42,202
0.76	Q53H37	Calmodulin-like protein 5	CALML5	4	15,876
0.73	Q5QNW6	Histone H2B type 2-F	HIST2H2BF	5	13,920
0.70	A0A024R6I3	Transmembrane emp24 domain-containing protein 10	TMED10	3	24,976
0.70	Q71UI9	Histone H2A.V	H2AFV	4	13,509
0.70	Q7Z7M4	Superoxide dismutase; mitochondrial	SOD2	5	23,672
−0.81	F6XU50	Lymphoid-specific helicase	HELLS	6	82,732
−0.82	B4DTD8	Glypican-3; secreted glypican-3	GPC3	3	63,727
−0.83	A7BI36	Ribosome-binding protein 1	RRBP1	83	165,750
−0.83	A0A1L5BXV2	Receptor expression-enhancing protein	REEP6	2	20,733
−0.87	Q53G72	B-cell receptor-associated protein 31	BCAP31	4	27,931
−1.11	B3KPF6	Striatin-4	STRN4	3	38,960
−1.20	Q96IF1	LIM domain-containing protein ajuba	AJUBA	5	56,933
−1.46	B4DZM3	Ribosomal RNA processing protein 1	RRP1	3	46,150

**Table 2:** Canonical pathways modulated in NAFLD versus control cells.

Ingenuity Canonical Pathways	*p*-Value	z-Score	Molecules
Unfolded protein response	1.32 × 10^−6^	−2.44	CALR,HSP90B1,HSPA5,P4HB,PDIA6,UBXN4
Protein kinase A signaling	9.12 × 10^−4^	−2.33	CALML5,H1-2,H1-4,H1 5,MYH10,MYL12A,PDIA3,PRKAR1A,RHOA
Xenobiotic metabolism CAR signaling pathway	3.09 × 10^−2^	−2	ALDH3A2,GSTZ1,HSP90B1,MGST1
HER-2 signaling in breast cancer	3.31 × 10^−2^	−2	ARF4,COX6B1,RPS6,YES1
Insulin secretion signaling pathway	6.61 × 10^−2^	−2	PDIA3,PRKAR1A,SSR4,YES1
Xenobiotic metabolism PXR signaling pathway	2.04 × 10^−4^	−1.89	ALDH3A2,CES1,CES2,GSTZ1,HSP90B1,MGST1,PRKAR1A
Sirtuin signaling pathway	3.55 × 10^−2^	−1.34	H1-2,H1-4,H1-5,PRKDC,SOD2
Cardiac hypertrophy signaling (enhanced)	1.98 × 10^−1^	−1.34	CALML5,PDIA3,PRKAR1A,RHOA,RPS6
Xenobiotic metabolism AHR signaling pathway	1.95 × 10^−3^	−1	ALDH3A2,GSTZ1,HSP90B1,MGST1
Opioid signaling pathway	6.92 × 10^−2^	−1	AP1B1,CALML5,PRKAR1A,YES1
Integrin signaling	1.07 × 10^−2^	−0.44	ACTG1,ARF4,CAPNS1,MYL12A,RHOA
Cardiac hypertrophy signaling	1.74 × 10^−2^	−0.44	CALML5,MYL12A,PDIA3,PRKAR1A,RHOA
Synaptogenesis signaling pathway	4.57 × 10^−2^	−0.44	AP1B1,CALML5,PRKAR1A,RHOA,YES1
Estrogen receptor signaling	1.07 × 10^−3^	−0.37	HNRNPD,HSP90B1,MYL12A,PDIA3,PRKAR1A,PRKDC,RHOA,SOD2
ILK signaling	6.76 × 10^−3^	0.44	ACTG1,KRT18,MYH10,MYH9,RHOA
Actin cytoskeleton signaling	1.38 × 10^−2^	0.44	ACTG1,MYH10,MYH9,MYL12A,RHOA
Hepatic fibrosis signaling pathway	9.55 × 10^−3^	1.13	CALML5,MYL12A,PRKAR1A,RHOA,SOD2,TFRC,YAP1
Ferroptosis signaling pathway	1.38 × 10^−4^	1.63	ARF4,FDFT1,H2AX,SLC39A14,TFRC,YAP1

## Data Availability

The data presented in this study are available in the Supplementary Materials.

## Supplementary Materials

The following supporting information can be downloaded at: https://www.mdpi.com/article/10.3390/ijms23169025/s1.
